# Genome-wide association study identifies novel loci and candidate genes for rust resistance in wheat (*Triticum aestivum* L.)

**DOI:** 10.1186/s12870-024-05124-2

**Published:** 2024-05-17

**Authors:** Hanif Khan, Gopalareddy Krishnappa, Sudheer Kumar, Narayana Bhat Devate, Nagenahalli Dharmegowda Rathan, Satish Kumar, Chandra Nath Mishra, Sewa Ram, Ratan Tiwari, Om Parkash, Om Parkash Ahlawat, Harohalli Masthigowda Mamrutha, Gyanendra Pratap Singh, Gyanendra Singh

**Affiliations:** 1 ICAR–Indian Institute of Wheat and Barley Research, Karnal, 132001 India; 2 ICAR–Sugarcane Breeding Institute, Coimbatore, 641007 India; 3 International Centre for Agriculture Research in the Dry Area - Food Legume Research Platform, Amlaha, MP 466113 India; 4 Corteva Agriscience, Hyderabad, Telangana 500081 India; 5 ICAR–National Bureau of Plant Genetic Resources, New Delhi, 110012 India

**Keywords:** Wheat rust, GWAS, SNP, Linkage disequilibrium, Stripe rust, Stem rust

## Abstract

**Background:**

Wheat rusts are important biotic stresses, development of rust resistant cultivars through molecular approaches is both economical and sustainable. Extensive phenotyping of large mapping populations under diverse production conditions and high-density genotyping would be the ideal strategy to identify major genomic regions for rust resistance in wheat. The genome-wide association study (GWAS) population of 280 genotypes was genotyped using a 35 K Axiom single nucleotide polymorphism (SNP) array and phenotyped at eight, 10, and, 10 environments, respectively for stem/black rust (SR), stripe/yellow rust (YR), and leaf/brown rust (LR).

**Results:**

Forty-one Bonferroni corrected marker-trait associations (MTAs) were identified, including 17 for SR and 24 for YR. Ten stable MTAs and their best combinations were also identified. For YR, *AX-94990952* on 1A + *AX-95203560* on 4A + *AX-94723806* on 3D + *AX-95172478* on 1A showed the best combination with an average co-efficient of infection (ACI) score of 1.36. Similarly, for SR, *AX-94883961* on 7B + *AX-94843704* on 1B and *AX-94883961* on 7B + *AX-94580041* on 3D + *AX-94843704* on 1B showed the best combination with an ACI score of around 9.0. The genotype PBW827 have the best MTA combinations for both YR and SR resistance. In silico study identifies key prospective candidate genes that are located within MTA regions. Further, the expression analysis revealed that 18 transcripts were upregulated to the tune of more than 1.5 folds including 19.36 folds (TraesCS3D02G519600) and 7.23 folds (TraesCS2D02G038900) under stress conditions compared to the control conditions. Furthermore, highly expressed genes in silico under stress conditions were analyzed to find out the potential links to the rust phenotype, and all four genes were found to be associated with the rust phenotype.

**Conclusion:**

The identified novel MTAs, particularly stable and highly expressed MTAs are valuable for further validation and subsequent application in wheat rust resistance breeding. The genotypes with favorable MTA combinations can be used as prospective donors to develop elite cultivars with YR and SR resistance.

**Supplementary Information:**

The online version contains supplementary material available at 10.1186/s12870-024-05124-2.

## Introduction

Wheat (*Triticum* sp.) is the most widely cultivated and traded cereal worldwide [1]. Globally, consumption of wheat based food products is increasing due to changed dietary patterns driven by urbanization and rising income [2]. To achieve the required quantity of wheat production by 2050, the annual mean yield needs to increase from the present level of 1.2% to the tune of 1.6% [3, 4]. Recent crop improvement technologies including marker assisted selection (MAS), SpeedGS (speed breeding + genomic selection), and genome editing (GE) will complement the conventional crop improvement approaches to enhance the genetic gains in crop plants [5]. Also, major research efforts are required to safeguard wheat production against biotic and abiotic stresses.

Globally, all three wheat rusts i.e., stem/black rust (SR), stripe/yellow rust (YR), and leaf/brown rust (LR) caused by *Puccinia striiformis* f. sp. *tritici* (*Pst*), *Puccinia graminis* f. sp. *tritici* (*Pgt*) and *Puccinia triticina* (*Pt*), respectively are important fungal diseases. The YR causes frequent crop loss in the range of 0.1 to 5.0% based on the varietal reaction and environmental conditions, the damage may increase to 25% [6], further, 100% crop loss may occur under severe incidences [7]. The *Pst* fungus is widely distributed across the globe, which resulted in several YR epidemics in major wheat growing regions [8, 9] including Central and South Asia [10, 11]. Historically, YR occurrence is mostly restricted to cool weather conditions, however, the advent of the novel racial composition of the pathogen is slowly adapting to the elevated temperature, resulting in the spreading of the disease to non conventional areas [12, 13]. Similarly, SR is another destructive fungal disease with the potential to cause 100% yield loss on susceptible cultivars [14]. The LR is relatively less destructive than SR and YR, however, it is more widespread, as it has a high frequency of occurrences and wide distribution across the globe [15].

The genetics of wheat rust resistance is broadly grouped into two types; one is all stage resistance (ASR) genes which are generally race-specific and the second is adult plant resistance (APR) genes, also called partial resistance or slow rusting [16] which is generally race-nonspecific resistance. Genes involved in adequate levels of race-nonspecific resistance may have small to intermediate effects [17]. This kind of resistance manifests in plants that are susceptible at seedling stage but resistant once they reach the post seedling phases of development. This feature is called slow rusting and is frequently associated with some forms of APR [18]. At present, 86 genes have been identified and catalogued for YR resistance in wheat [19, 20] and most of the identified genes are found to be race-specific. Similarly, a total of 83 major genes have been identified and catalogued for LR resistance [20, 21]. The majority of the leaf/brown rust resistance (*Lr*) genes confer ASR, while 14 genes induce APR reaction [20, 22, 23]. A total of 63 genes are catalogued for SR resistance [20, 24]. Most of the identified stem/black rust resistance (*Sr*) genes are seedling resistance genes, and only six genes viz., *Sr2, Sr55, Sr56, Sr57, Sr58*, and *Sr63* confer APR response to the SR pathogen [20, 25]. The majority of the identified rust resistance genes of all three rusts were introgressed from related species except a few genes, which were identified in bread wheat.

The cultivars with single gene based resistance will break down under severe disease pressure conditions [26]. Hence, the combination of diverse APR genes with one or few ASR genes is necessary to develop durable resistance [27, 28]. Although major gene based rust resistance through the deployment of novel genes is important; critical also the minor gene based resistance through quantitative trait loci (QTL) mapping. QTL mapping and GWAS are two common methods to dissect complex disease traits. The QTL mapping has many limitations including large population, development time, limited resolution caused by few crossover events, and comparatively less polymorphism [29]. GWAS uses diverse populations or genotypes with different geographical origins [30], hence, requires less time and resources as there is no need to perform controlled crosses to develop mapping populations unlike in QTL mapping. GWAS utilizes populations that have undergone many historical and ancestral recombination events since domestication and therefore have higher resolution. Additionally, diverse germplasm captures superior alleles that have been missed by routine breeding. GWAS is based on the linkage disequilibrium (LD) that formed over the generations and the genomic regions harboring QTLs can be detected even in the absence of the inclusion of causal mutations among the set of available molecular markers [31]. GWAS is becoming more relevant to dissect quantitative traits in complex genomes like wheat, particularly in the era of next-generation sequencing (NGS), which resulted in the development of several high throughput SNP arrays [32–34]. Previously, different genetic panels and marker systems were used to identify marker-trait associations (MTAs) through GWAS analysis for YR field resistance [12, 35–44]. Similarly, several MTAs were identified for SR resistance through GWAS [12, 45–51]. Also, GWAS studies identified MTAs for LR resistance [52–56]. Although several MTAs were identified in various GWAS studies for wheat rust, the possibility of false positive occurrences is significantly higher, as most of the studies fixed lower significant threshold values (− log10 *p.value* 3.0–4.0), and also phenotyping was done in limited environments/production conditions. Hence, the determination of the optimum *p.value* threshold and conducting multi-environment evaluations with a large number of environments are very important to reduce the false positives and to obtain stable MTAs to deploy in MAS. Also, more genetic studies with diverse panels and marker systems along with multi-environment phenotyping may yield further novel and consistent genomic regions [57].

The recent developments in DNA technologies and reduced genotyping costs made genetic dissection of complex traits more accessible and effective. Further, the availability of wheat reference genome [58] has facilitated the identification of precise QTL positions and underlying candidate genes. Hence, this study was designed to (i) evaluate the genetically diverse wheat population for YR, SR, and LR resistance in multi-environments; (ii) conduct GWAS analysis for rust resistance to identify the MTAs to deploy in MAS; (iii) identify the putative candidate genes associated with the MTAs. Forty-one Bonferroni corrected MTAs including 10 stable MTAs and their best combinations were identified. PBW827 has the best MTA combinations for both YR and SR resistance. In silico study identified key putative candidate genes and expression analysis revealed 18 transcripts were upregulated to the tune of more than 1.5 folds under stress conditions compared to the control conditions.

## Results

### SNP distribution

Among the different marker systems, SNPs are abundant and have genome-wide distribution and hence well suited for GWAS. Similarly, among different SNP genotyping methods, hybridization based chip genotyping yields good quality data returns with fewer missing values. A 35 K mid density markers were used as it is a subset of important markers from the high density set. A total of 14,790 curated markers that are qualified with stringent quality checks were selected for further GWAS analysis. These curated informative markers and respective rust data were used for MTA identification. Subgenome wise, 5649 SNPs were identified on subgenome B, 4590 SNPs on subgenome D, and 4551 SNPs on subgenome A. Chromosome wise distribution of SNPs on A subgenome are as follows: 751 (1A), 756 (2A), 587 (3A), 493 (4A), 699 (5A), 515 (6A), 750 (7A); similarly, chromosome wise distribution on B subgenome revealed that 1077 SNPs on 1B, 992 (2B), 726 (3B), 465 (4B), 863 (5B), 766 (6B), 760 (7B). Subgenome D marker distribution is as follows: 986 (1D) 951 (2D), 648 (3D), 264 (4D), 657 (5D), 459 (6D),625 (7D).

### Population structure and LD

The details of the principal component analysis (PCA), kinship matrix of the GWAS population, and LD plots are given in our earlier published report [59]. Briefly, the squared correlation coefficient (*r*^*2*^) for all the SNPs was calculated and plotted against the genetic distance in the base pair (bp) to estimate the LD values. Subgenome A had an LD decay of 3.6 centimorgan (cM) and the decay was rapid, the LD distance of 5.2 cM was recorded for the D subgenome, and 5.7 cM for subgenome B; similarly, an LD decay of 4.9 cM was recorded for the whole genome and further details are presented in our earlier report [59].

### GWAS analysis and MTAs identification

**Fig. 1:**
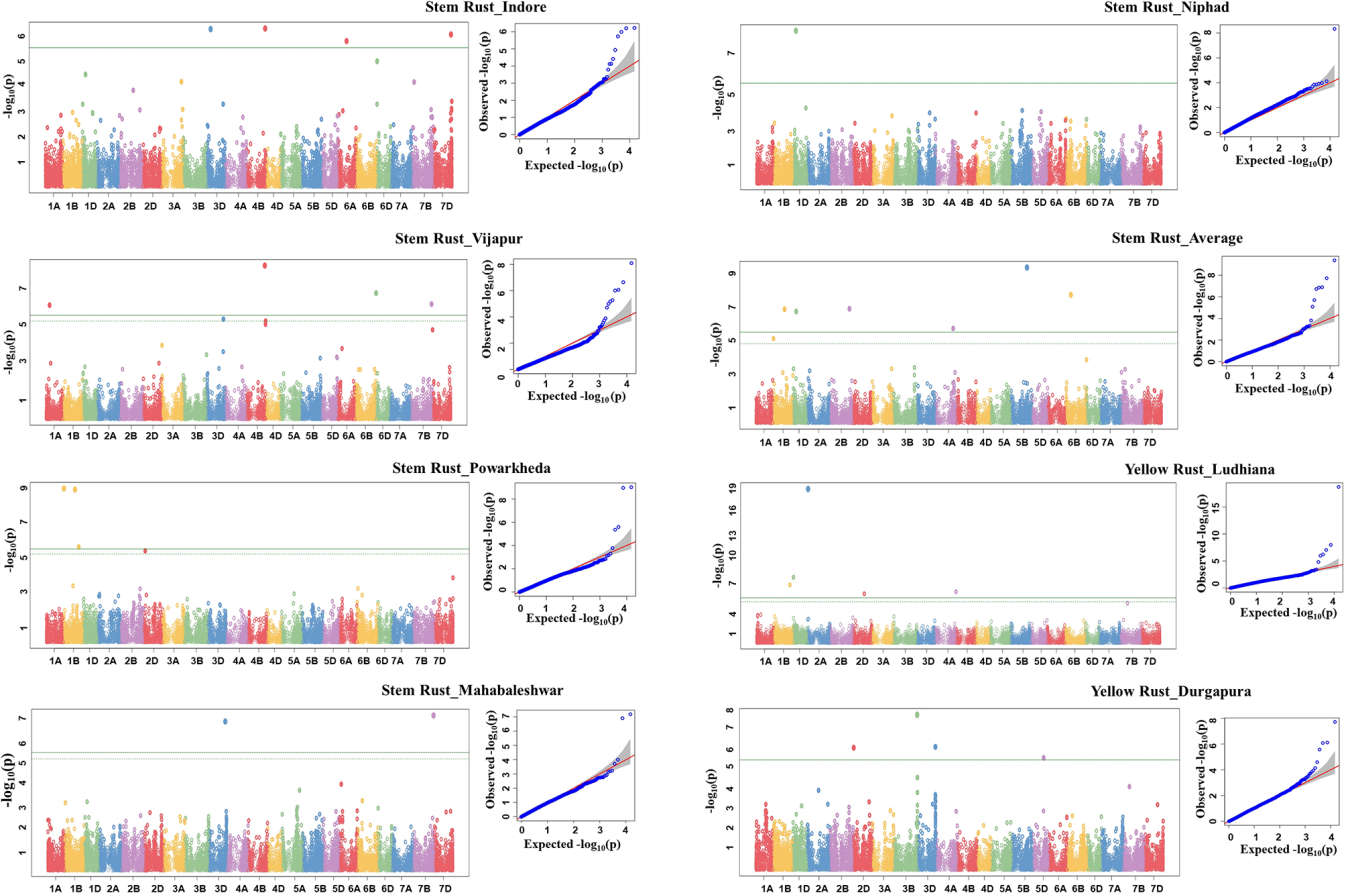
Manhattan and QQ plots for stem rust at Indore, Vijapur, Powarkheda, Mahabaleshwar, Niphad, Average and yellow rust at Ludhiana and Durgapura locations. The X-axis in Manhatten plots indicates the name of the chromosome

**Fig. 2:**
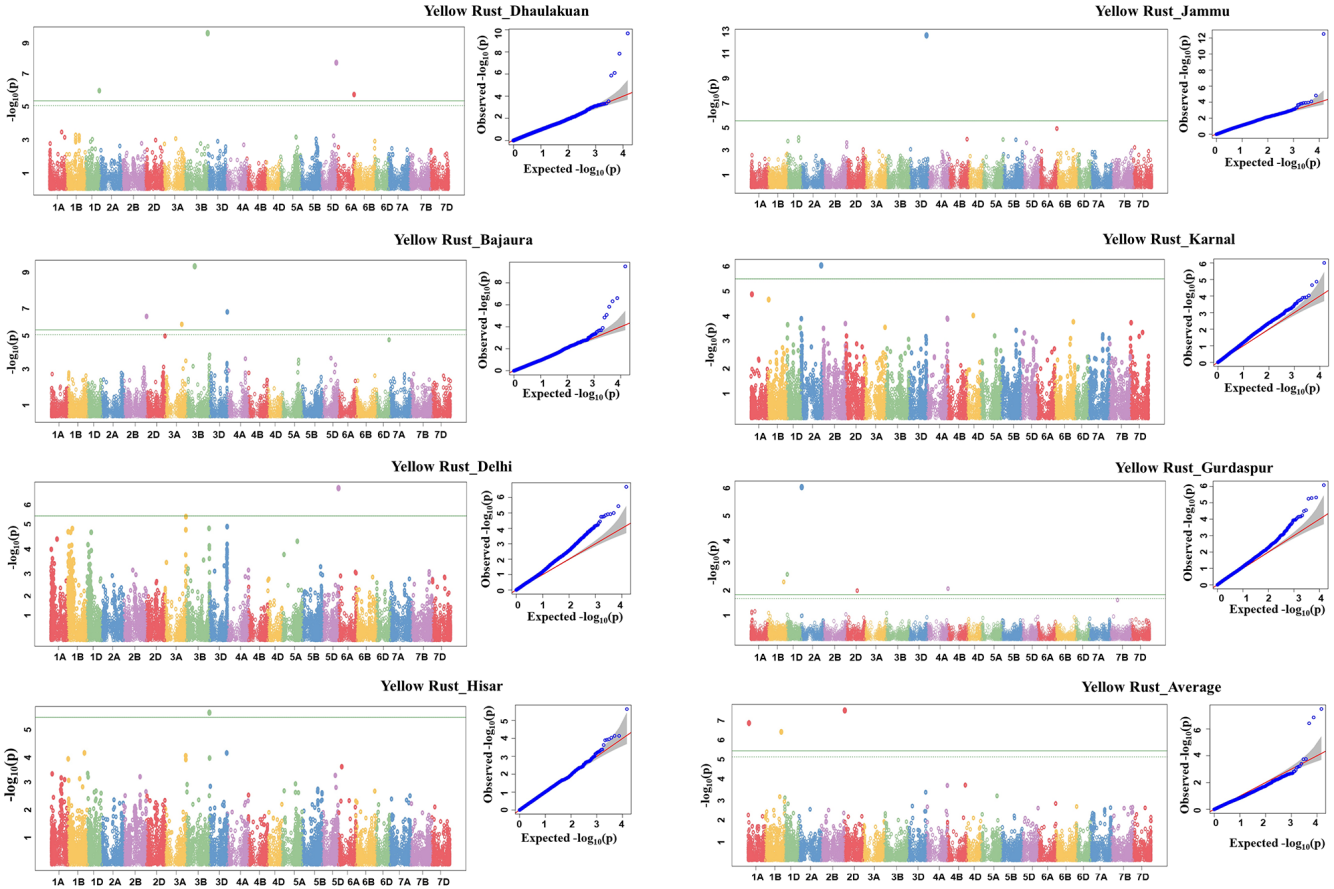
Manhattan and QQ plots for yellow rust at Dhaulakuan, Bajaura, Delhi, Hisar, Jammu, Karnal, Gurdaspur locations along with the average. The X-axis in Manhatten plots indicates the name of the chromosome

Forty-one Bonferroni corrected MTAs for SR and YR were identified (Table 1; Figs. 1 and 2). Also depicted (Figs. 1 and 2) are QQ plots to represent the observed vs. expected associations of SNPs. Although 16 MTAs were identified for LR at a significant threshold of (− log10 *p.value* ≥ 4.0), none of them could qualify to be identified at the threshold level of the Bonferroni correction factor, which is much higher. Twenty-four novel MTAs identified for YR and 17 MTAs identified for SR. Similarly, 17, 14, and 10 MTAs were identified on D, B, and A subgenomes, respectively. Further, 10 stable MTAs that are identified in more than one environment were identified, including five each for SR and YR.

**Table 1:** The list of identified MTAs for stem and stripe rust

Trait	Environment	MTA	Chr.	Position (Mb)	*p*. value	PVE (%)	Known APR gene	
SR	Indore	*AX-95168938*	6A	288.4	1.86E-06	2.8	-	
		*AX-94878781*	4B	645.3	5.95E-07	4.0	-	
		*AX-94472028*	3D	118.5	6.26E-07	3.2	-	
		*AX-94973922*	7D	597.0	1.02E-06	7.0	*Sr57*	
	Vijapur	*AX-94575656*	1A	119.7	9.83E-07	12.2	-	
		*AX-94878781*	4B	645.3	7.97E-09	1.4	-	
		*AX-94883961*	7B	717.0	8.61E-07	9.4	-	
		*AX-94751325*	6D	3.0	2.27E-07	4.0	-	
	Powarkheda	*AX-94716205*	1B	12.8	9.64E-10	6.9	*Sr58*	
		*AX-94843704*	1B	395.3	1.11E-09	11.9	-	
		*AX-94916753*	1B	524.2	2.57E-06	9.5	-	
	Mahabaleshwar	*AX-94883961*	7B	717.0	6.52E-08	17.4	-	
		*AX-94580041*	3D	567.2	1.25E-07	14.4	-	
	Niphad	*AX-94543538*	1D	81.3	4.72E-09	19.4	-	
	Average	*AX-94641391*	4A	628.5	2.02E-06	1.3	-	
		*AX-94843704*	1B	395.3	1.40E-07	2.6	-	
		*AX-94664270*	2B	680.4	1.30E-07	6.9	-	
		*AX-94421372*	5B	550.2	4.24E-10	6.4	*Sr56*	
		*AX-95084685*	6B	177.3	1.90E-08	9.2	-	
		*AX-94691001*	1D	110.7	1.90E-07	2.6	-	
YR	Ludhiana	*AX-95148952*	2A	14.8	1.93E-19	6.0	*Yr86*	
		*AX-95203560*	4A	743.9	6.09E-07	4.4	-	
		*AX-94875635*	1B	564.9	9.04E-08	1.7	*Yr29*	
		*AX-94762873*	1D	0.6	1.06E-08	13.7	-	
		*AX-95256011*	2D	388.8	1.07E-06	2.4	*Yr16, Yr54*	
	Durgapura	*AX-94872685*	3B	804.7	1.92E-08	20.4	*Yr30, Yr58, Yr80*	
		*AX-94734286*	2D	16.4	8.38E-07	12.1	*Yr16, Yr54*	
		*AX-94769906*	3D	602.3	7.62E-07	19.6	*Yr49, Yr71*	
		*AX-94448814*	5D	376.2	2.67E-06	8.8	-	
	Dhaulakuan	*AX-94990952*	6A	607.7	1.38E-06	2.6	-	
		*AX-95107273*	3B	810.3	2.13E-10	8.4	*Yr30, Yr58*	
		*AX-94590703*	1D	470.6	7.94E-07	11.8	-	
		*AX-94408063*	5D	546.9	1.45E-08	4.2	-	
	Bajaura	*AX-94681852*	3A	584.7	1.50E-06	5.8	-	
		*AX-94480089*	2B	797.3	4.75E-07	1.8	-	
		*AX-94938276*	3B	290.8	3.21E-10	13.6	*Yr30, Yr58*	
		*AX-94723806*	3D	607.1	2.48E-07	4.4	*Yr49, Yr71*	
	Delhi	*AX-94561441*	5D	558.2	2.02E-07	21.0	-	
	Hisar	*AX-94390305*	3B	814.0	2.27E-06	18.6	*Yr30, Yr58*	
	Jammu	*AX-94513116*	3D	563.9	3.15E-13	19.8	*Yr49, Yr71*	
	Karnal	*AX-94,476,121*	2A	704.8	1.00E-06	16.8	*Yr86*	
	Gurdaspur	*AX-95165557*	2A	4.0	8.37E-07	18.9	*Yr86*	
	Average	*AX-95172478*	1A	33.0	1.39E-07	14.7	-	
		*AX-94875635*	1B	564.9	3.81E-07	4.2	*Yr29*	
		*AX-94690433*	2D	14.3	3.23E-08	3.0	*Yr16, Yr54*	

SR: stem/black rust; YR: yellow/stripe rust; MTA: market-trait associations; Chr.: chromosome; Mb: megabase; PVE: phenotypic variation explained

### MTAs for SR

Seventeen MTAs for SR resistance were identified, including 3 MTAs on 1A, 4A, and 6A; 8 MTAs on 1B, 2B, 4B, 5B, 6B, and 7B; and 6 MTAs on 1D, 3D, 6D, and 7D. The phenotypic variation explained (PVE) by different MTAs of SR resistance ranged from 1.4% (*AX-94878781* at Vijapur) to 19.4% (*AX-94543538* at Niphad). Out of 17 MTAs, *AX-94575656* (1 A), *AX-94843704* (1B), *AX-94883961* (7B), *AX-94580041* (3D), and *AX-94543538* (1D) explained ≥ 10.0% PVE, and mapped at 119.7 Mb, 395.3 Mb, 717.0 Mb, 567.2 Mb, 81.3 Mb, respectively. Subgenome wise distribution of QTLs suggests that more than 50% were located on subgenome B alone. These include *AX-94421372* (5B), *AX-94664270* (2B), *AX-94716205* (1B), *AX-94843704* (1B), *AX-94878781* (4B), *AX-94883961* (7B), *AX-94916753* (1B), *AX-95084685* (6B) located at 550.2 Mb, 680.4 Mb, 12.8 Mb, 395.3 Mb, 645.3 Mb, 717 Mb, 524.2 Mb, and 177.3 Mb, respectively. Three APR genes namely *Sr57*, *Sr58*, and *Sr56* were located on 7D, 1B, and 5B chromosomes, respectively harbor MTAs i.e., *AX-94973922*, *AX-94716205*, and *AX-94421372* at 597.0 Mb, 12.8 Mb, and 550.2 Mb on the same chromosomes.

### MTAs for YR

Twenty-five MTAs were identified for YR, including seven MTAs on the A subgenome (1A, 2A, 3A, 4A, 6A), seven MTAs on the B subgenome (1B, 2B, 3B), and 11 MTAs on the D subgenome (1D, 2D, 3D, 5D). The PVE by different QTLs ranged from 1.7% (*AX-94875635*) to 21.0% (*AX-94561441*). A maximum of five MTAs were mapped in the Ludhiana environment and four MTAs each in Durgapura, Dhaulakuan, and Bajaura environments; the remaining five environments i.e., Karnal, Jammu, Hisar, Gurdaspur, and Delhi had one MTA each along with three MTAs for average mean. Out of 25 MTAs, *AX-94561441* (5D), *AX-94872685* (3B), *AX-94513116* (3D), *AX-94769906* (3D), *AX-95165557* (2A), *AX-94390305* (3B), *AX-94476121* (2A), *AX-95172478* (1A), *AX-94762873* (1D), *AX-94938276* (3B), *AX-94734286* (2D), and *AX-94590703* (1D) explained ≥ 10.0% PVE and mapped at 558.2 Mb, 804.7 Mb, 563.9 Mb, 602.3 Mb, 4.0 Mb, 814.0 Mb, 704.8 Mb, 33.0 Mb, 0.6 Mb, 290.8 Mb, 16.4 Mb, 470.6 Mb, respectively. Some of the important YR APR genes including *Yr16*, *Yr29*, *Yr30*, *Yr49*, *Yr54*, *Yr58*, *Yr71*, *Yr80*, and *Yr86* were also located on the same chromosomes where the MTAs were identified.

### Stable MTAs

Ten stable MTAs were identified, including five each for SR and YR resistance, and the details are given in Table 2. For YR, *AX-94990952* on 6 A chromosome mapped at 607.7 Mb was identified in three environments viz., Gurdaspur, Dhaulakuan, and Jammu, which explained PVE of 4.2%, 2.6%, and 4.8%, respectively. Further, *AX-94723806* on 3D and *AX-95203560* on 4 A were located at 607.1 Mb and 743.9 Mb, respectively were identified in two environments (Bajaura and Delhi for *AX-94723806* with PVE of 4.4% and 4.2%, respectively) and (Ludhiana and Gurdaspur for *AX-95203560* with PVE of 4.4% and 5.2%, respectively). The remaining two stable MTAs viz., *AX-94,875635* (PVE of 1.7% and 4.2%) and *AX-95172478* (PVE of 4.9% and 14.7%) mapped at 564.9 Mb and 33.0 Mb were identified in one environment along with the average mean. For SR, two MTAs viz., *AX-94580041* (3D) and *AX-94883961* (7B) located at 567.2 Mb and 717.0 Mb were identified in Mahabaleshwar and Vijapur environments and the PVE ranged between 9.4 and 17.4%.

**Table 2:** The list of stable MTAs for stem/black rust (5 MTAs) and yellow/stripe rust (5 MTAs)

Trait	MTA	Chr.	Position (Mb)	Environment	*p*.value	PVE (%)
SR	*AX-94878781*	4B	645.3	Indore	5.95E-07	4.0
				Vijapur	7.97E-09	1.4
	*AX-94580041*	3D	567.2	Mahabaleshwar	1.25E-07	14.4
				Vijapur	5.34E-06	9.6
	*AX-94883961*	7B	717.0	Mahabaleshwar	6.52E-08	17.4
				Vijapur	8.61E-07	9.4
	*AX-94716205*	1B	12.8	Powarkheda	9.64E-10	6.9
				Average	8.19E-06	5.1
	*AX-94843704*	1B	395.3	Powarkheda	1.11E-09	11.9
				Average	1.40E-07	2.6
YR	*AX-94990952*	6A	607.7	Gurdaspur	6.85E-05	4.2
				Dhaulakuan	1.38E-06	2.6
				Jammu	1.47E-05	4.8
	*AX-94723806*	3D	607.1	Bajaura	2.48E-07	4.4
				Delhi	5.99E-05	4.2
	*AX-95203560*	4A	743.9	Ludhiana	6.09E-07	4.4
				Gurdaspur	5.74E-06	5.2
	*AX-94875635*	1B	564.9	Ludhiana	9.04E-08	1.7
				Average	3.81E-07	4.2
	*AX-95172478*	1A	33.0	Karnal	1.36E-05	4.9
				Average	1.39E-07	14.7

SR: stem/black rust; YR: yellow/stripe rust; MTA: market-trait associations; Chr.: chromosome; Mb: megabase; PVE: phenotypic of variation explained

**Fig. 3:**
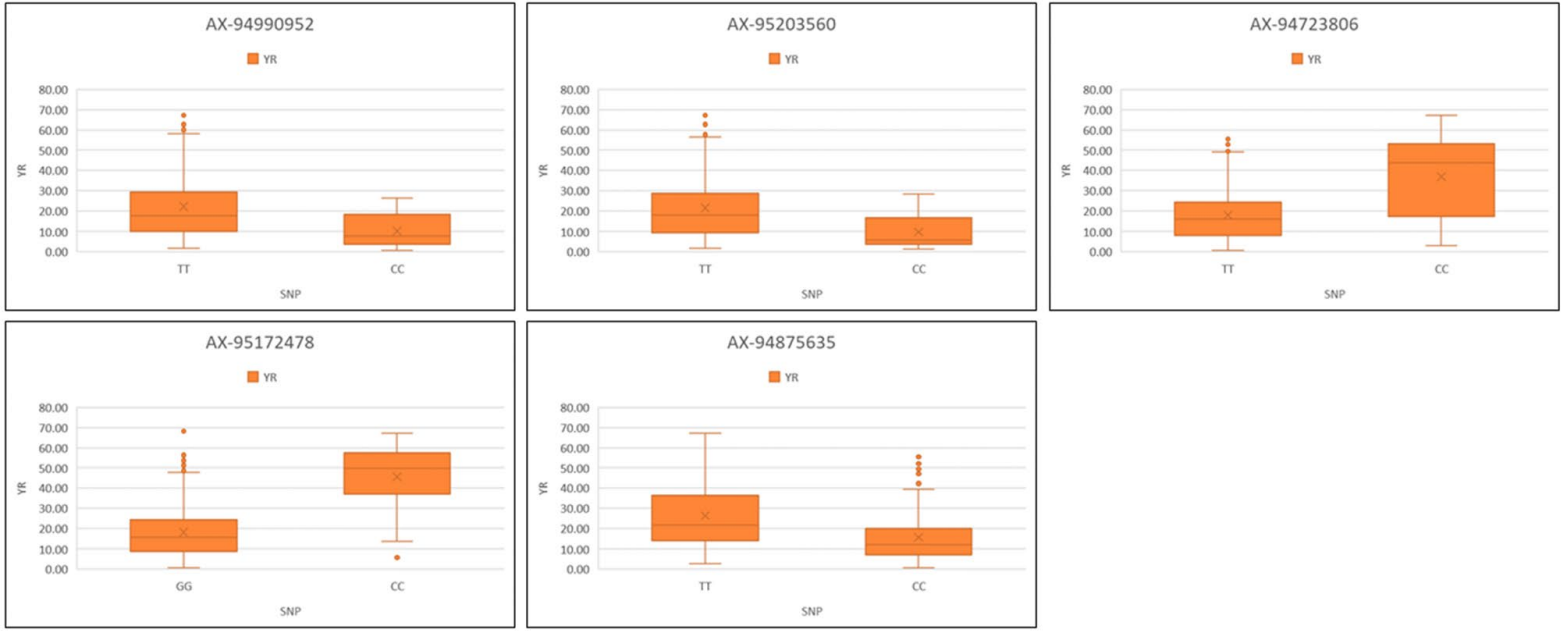
Allelic differences of the stable MTAs identified for stem rust resistance in the GWAS population. SR: Stem/black rust. Significance at *p* < 0.01 between the alleles

**Fig. 4:**
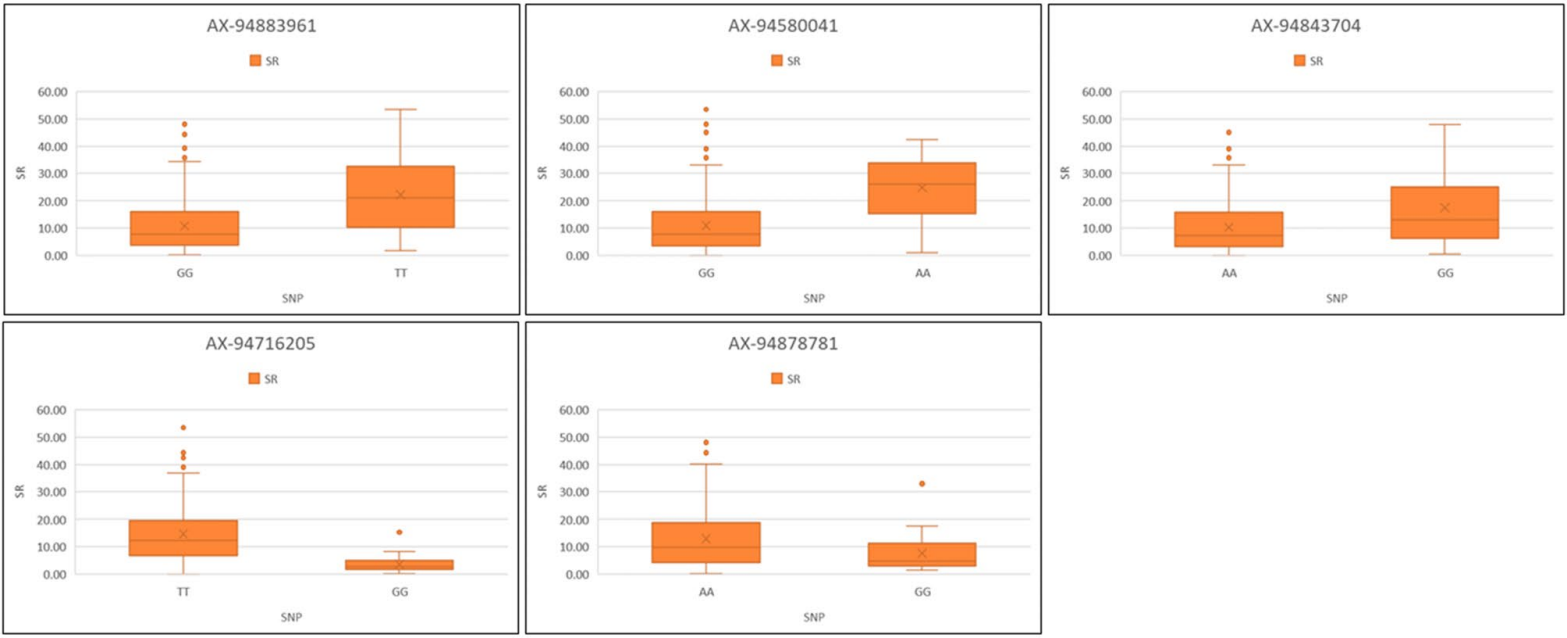
Allelic differences of the stable MTAs identified for stem rust resistance in the GWAS population. SR: Stem/black rust. Significance at *p* < 0.01 between the alleles

One MTA i.e., *AX-94878781* (4B) mapped at 645.3 Mb was identified in two environments (Indore and Vijapur). Similarly, *AX-94716205* and *AX-94843704* located on 1B chromosome were mapped at 12.8 Mb and 395.3 Mb were identified in Powarkheda along with average mean with PVE ranging from 2.6 to 11.9%. The box plots in Figs. 3 and 4 indicate the allelic differences of stable MTAs for SR and YR, respectively. For SR, Alleles G, G, A, G, and G were superior for consistent MTAs i.e., *AX-94883961*, *AX-94580041*, *AX-94843704*, *AX-94716205*, and *AX-94878781*, respectively. For YR, C, C, T, G, and C were superior for consistent MTAs i.e., *AX-94990952*, *AX-95203560*, *AX-94723806*, *AX-95172478*, and *AX-94875635*, respectively.

### Combination effect of stable MTAs

The phenotypic effects of the combination of stable MTAs were investigated for YR and SR (Table 3). For YR, *AX-94990952* on 6A, *AX-95203560* on 4A, *AX-94723806* on 3D, and *AX-95172478* on 1A had the largest effect individually in the desirable direction (lower ACI values are the desirable ones) with ACI values of 10.25, 9.78, 18.10, and 18.22, respectively. *AX-94990952* on 6A + *AX-95203560* on 4A + *AX-94723806* on 3D + *AX-95172478* on 1 A showed the best combination with an ACI score of 1.36 and is present in only one genotype i.e., PBW827. The next best combination was *AX-94990952* on 6A + *AX-95203560* on 4A with an ACI score of 3.52 and present in MP3529 and PBW827 genotypes. The other best combination includes *AX-95203560* on 4A + *AX-95172478* on 1A with an ACI score of 8.14 and is present in six genotypes (DBW310, HUW839, PBW827, PBW841, WH1274, and WH1283). For SR, *AX-94883961* on 7B, *AX-94580041* on 3D, and *AX-94843704* on 1B had the largest effect individually in the desirable direction (lower ACI values are the desirable ones) with ACI values of 10.77, 10.87, and 10.30, respectively and there is no discernible increase when the additional MTAs were combined. Although *AX-94883961* on 7B + *AX-94843704* on 1B had the ACI score of 9.45, *AX-94883961* on 7B + *AX-94580041* on 3D + *AX-94843704* on 1B combination had the ACI score of 9.49 and present in 181 and 180 genotypes, respectively. Most importantly, the genotype PBW827 had the best QTL combinations for both YR and SR resistance.

**Table 3:** Combination effect of stable MTAs

Trait	MTA	Allele Call	No. of Genotypes	Rust Score (ACI)	Genotypes
SR	*AX-94883961* (7B, 716.96 Mb)	T	21	22.17	**-**
		G	239	10.77	**-**
	*AX-94,80041* (3D, 567.19 Mb)	A	8	24.88	**-**
		G	237	10.87	**-**
	*AX-94843704* (1B, 395.27 Mb)	A	206	10.30	**-**
		G	25	17.43	**-**
	*AX-94883961* + *AX-94580041*	G + G	209	10.03	DBW320, HPW473, WH1274
	*AX-94883961* + *AX-94843704*	G + A	181	9.45	**-**
	*AX-94580041* + *AX-94843704*	G + A	204	10.36	**-**
	*AX-94883961* + *AX-94580041* + *AX-94843704*	G + G + A	180	9.49	HD3348, HS676, HS679, PBW813, PBW827, RVW4301, HI1655
YR	*AX-94990952* (6A, 607.68 Mb)	C	11	10.25	**-**
		T	236	22.23	**-**
	*AX-95203560* (4A, 743.92 Mb)	C	9	9.78	**-**
		T	232	21.61	**-**
	*AX-94723806* (3D, 607.09 Mb)	C	50	36.93	**-**
		T	182	18.10	**-**
	*AX-95172478* (1A, 330.22 Mb)	C	28	45.62	**-**
		G	148	18.22	**-**
	*AX-94990952* + *AX-95203560*	C + C	2	3.52	MP3529, PBW827
	*AX-94990952* + *AX-94723806*	C + T	5	9.79	DBW312, DBW320, HPW473, PBW813, PBW827
	*AX-94990952* + *AX-95172478*	C + G	10	10.71	DBW312, DBW320, HD3348, HPW473, HS676, HS679, PBW813, PBW827, RVW4301, HI1655
	*AX-95203560* + *AX-94723806*	C + T	4	10.01	DBW310, HUW839, PBW827, WH1274
	*AX-95203560* + *AX-95172478*	C + G	6	8.14	DBW310, HUW839, PBW827, PBW841, WH1274, WH1283
	*AX-94723806* + *AX-95172478*	T + G	177	17.54	**-**
	*AX-94990952* + *AX-94723806* + *AX-95172478*	C + T + G	5	9.79	DBW312, DBW320, HPW473, PBW813, PBW827
	*AX-94990952* + *AX-95203560* + *AX-94723806* + *AX-95172478*	C + C + T + G	1	1.36	PBW827

SR: stem/black rust; YR: yellow/stripe rust; ACI: average coefficient of infection

The MTAs for SR and YR were used to locate the putative genes using the annotated wheat reference sequence (Wheat Chinese Spring IWGSC Ref Seq v2.1) genome assembly (2021)) and are listed in Table 4. The MTAs i.e., *AX-94716205*, *AX-94472028*, *AX-94878781*, *AX-94421372*, and *AX-94641391*, associated with SR were found to encode disease resistance protein in crop plants (TraesCS1B02G026300), ankyrin repeat (TraesCS3D02G152600), protein kinase, ATP binding site (TraesCS4B02G353600), protein kinase domain (TraesCS5B02G371800), serine/threonine-protein kinase (TraesCS4A02G353300), and START domain (TraesCS4A02G353400). Similarly, *AX-94390305*, *AX-94408063*, *AX-94448814*, *AX-94476121*, *AX-94734286*, *AX-94762873*, and *AX-95165557* associated with YR were found to encode leucine-rich repeat domain superfamily (TraesCS3B02G587400), serine-threonine/tyrosine-protein kinase (TraesCS5D02G531200), C2 domain (TraesCS5D02G273300), zinc finger, RING/FYVE/PHD-type (TraesCS2A02G456200), protein kinase domain (TraesCS2A02G456100), Myb/SANT-like domain (TraesCS2A02G455700), plant disease resistance protein (TraesCS2D02G044800), zinc finger, FYVE/PHD-type (TraesCS1D02G002400), leucine-rich repeat, typical subtype (TraesCS1D02G002700), and START domain (TraesCS2A02G010200).

**Table 4:** Putative candidate genes for stem/black rust and yellow/stripe rust

Trait	Env.	MTA		Chr.	Position (bp)	TraceID	Encoded Protein	Functions
SR	Powarkheda		*AX-94716205*	1B	12,811,607–12,814,290	TraesCS1B02G026300	Disease resistance protein, plants	Disease resistance in crop plants
	Indore		*AX-94472028*	3D	118,534,889–118,549,915	TraesCS3D02G152600	Ankyrin repeat	Ankyrin repeat and WRKY receptors regulate wheat stripe rust resistance [60]
	Indore		*AX-94878781*	4B	645,300,088–645,304,809	TraesCS4B02G353600	Protein kinase, ATP binding site	*Sr43* encoded protein kinase ATP binding site confers resistance to a wide range of isolates of the stem rust pathogen [61].
	Average		*AX-94421372*	5B	550,225,703–550,230,296	TraesCS5B02G371800	Protein kinase domain	*Sr60* driven protein with two kinase domains confers intermediate level of resistance to *Pgt* [62].
	Average		*AX-94641391*	4A	628,483,615–628,487,001	TraesCS4A02G353300	Serine/threonine protein kinase	Serine/threonine kinase gene i.e., *Rpg1* regulates stem rust resistance in barley [63].
					628,490,342–628,495,898	TraesCS4A02G353400	START domain	-
YR	Hisar		*AX-94390305*	3B	813,945,963–813,954,858	TraesCS3B02G587400	Leucine-rich repeat domain superfamily	Wheat rust genes encode proteins with NBS-LRR domains that confers resistance through hypersensitive cell death and high PR productions [64].
	Dhaulakuan		*AX-94408063*	5D	546,906,214–546,913,767	TraesCS5D02G531200	Serine threonine/tyrosine protein kinase	Serine/threonine kinase in WHEAT KINASE START1(WKS1) gene confers resistance to stripe rust [65].
	Durgapura		*AX-94448814*	5D	376,178,437–376,180,761	TraesCS5D02G273300	C2 domain	C2 domain protein-encoding gene i.e., *TaERG3* regulates stripe rust resistance in wheat [66].
	Karnal		*AX-94476121*	2A	704,785,260–704,789,592	TraesCS2A02G456200	Zinc finger, RING/FYVE/PHD type	Stripe rust resistance in wheat [67].
					704,772,997–704,777,010	TraesCS2A02G456100	Protein kinase domain	-
					704,758,041–704,762,734	TraesCS2A02G455700	Myb/SANT like domain	A novel R2R3-MYB transcription factor i.e., TaMYB29 confers stripe rust resistance in wheat [68]. MYB was the most abundant TF, which confers stripe rust resistance [69].
	Durgapura		*AX-94734286*	2D	16,344,385–16,345,554	TraesCS2D02G044800	Disease resistance protein, plants	Disease resistance in crop plants
	Ludhiana		*AX-94762873*	1D	568,769–574,727	TraesCS1D02G002400	Zinc finger, FYVE/PHD-type	*TaLSD1* is a negative regulator of programmed cell death and is involved in rust resistance against stripe rust pathogen [67].
					617,337–621,178	TraesCS1D02G002700	Leucine rich repeat, typical subtype	NBS-LRR protein activated by the pathogen effector protein enables the start of the defense response [70].
	Gurdaspur		*AX-95165557*	2A	3,964,835–3,968,074	TraesCS2A02G010200	START domain	START lipid binding domains in the WKS1 gene confers resistance to stripe rust in wheat [65]. A kinase and a putative START lipid binding domain are integral parts of the WKS1 gene which confers non race specific resistance to stripe rust [71].

Env.: environment; MTA: market-trait associations; Chr.: chromosome; bp: base pair

**Fig. 5:**
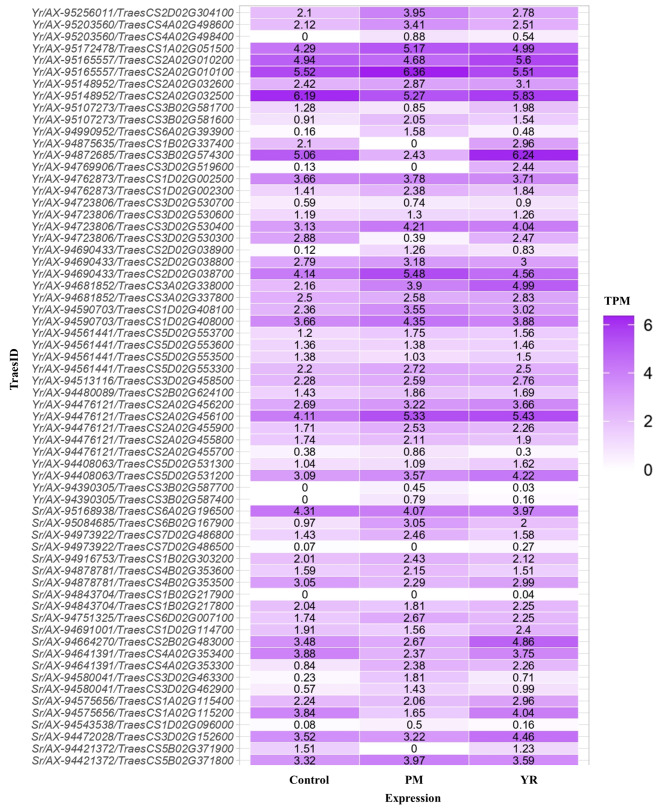
Heat map of expression analysis for the identified MTAs. PM: Powdery mildew; YR: Yellow rust

The putative genes associated with all 41 MTAs were used for expression analysis using wheat expression data, which revealed many transcripts whose expression levels are upregulated by many folds (Fig. 5 and Supplementary Table S2). Many of them have expressed under control as well as under disease stress. They were depicted in intense purple color in the heat map with respective transcription values (Fig. 5). However, 18 candidate genes were found to be overexpressed more than 1.5 folds under the disease stress. The two important candidate genes i.e., TraesCS3D02G519600 and TraesCS2D02G038900 expression levels were 19.36 and 7.23 fold higher under stress conditions. Similarly, TraesCS3D02G463300, TraesCS7D02G486500, and TraesCS6A02G393900 were found to be over expressed around 3 fold under disease stress condition. Further, four of the candidate genes viz., TraesCS3B02G587400, TraesCS3B02G587700, TraesCS1B02G217900, and TraesCS4A02G498400 were found to be expressed only under disease stress and no expression was observed under control condition. It is also notable that expression levels of these genes were also high under powdery mildew indicating the pleiotropic nature of the disease resistance. Highly expressed genes (TraesCS2D02G038900) and genes expressed only under stress conditions (TraesCS1B02G217900 and TraesCS4A02G498400) along with TraesCS3B02G587400 were subjected to find out the potential links to the rust phenotype. As a result, all four genes were found to be associated with rust resistance phenotype (Supplementary Fig. S1).

## Discussion

Wheat rust is important biotic stress, the development of rust resistant cultivars through conventional breeding complemented with marker based gene pyramiding is an ideal approach [72]. To date, three APR genes namely *Yr18/Lr34/Sr57*, *Yr36*, and *Yr46/Lr67/Sr55* have been cloned, among which the *Yr18* complex is extensively deployed [73, 74]. The ‘*Yr18* complex’ has been widely utilized in the CIMMYT bread wheat breeding program to give durable rust resistance [75]. The continuous evolution and quick dispersal of new rust pathotypes across the continents made it necessary to identify and utilize more durable sources of resistance [76]. It is desirable to use elite genetic backgrounds for mapping and introgression to avoid linkage drag [77]. Pyramiding of several resistance genes with additive effects into a single genetic background will aid in the prevention of the major breakdown of field resistance.

The multi-environment evaluation of the mapping population is crucial in identifying the stable MTAs to use in marker assisted breeding. In this direction, the GWAS population was phenotyped in eight environments for SR and 10 environments each for LR and YR. The details of rust resistant advanced breeding lines and check varieties are given in supplementary table S3. Out of 33 SR and LR (north) resistant genotypes, 11 are released varieties for various production conditions, and one genotype (DBW308) was registered as genetic stock for multiple disease resistance. Similarly, 20 advanced breeding lines and two check varieties (DBW316 and HI1654) exhibited high levels of YR and LR (north) resistance. All the genotypes are agronomically superior as they entered into the national varietal trial testing after several rounds of preliminary evaluations in different production conditions. One genotype i.e., PBW827 possesses all the favorable MTA combinations for YR and was highly resistant to both YR and LR (north). Hence, these resistant genotypes could be readily used in rust resistance breeding programmes.

Forty-one Bonferroni corrected MTAs were identified, including 17 for SR and 24 for YR resistance covering all three subgenomes. Five stable MTAs were identified each for SR i.e., *AX-94716205* and *AX-94843704* on chromosome 1B, *AX-94580041* on 3D, *AX-94878781* on 4B, *AX-94883961* on 7B and YR resistancei.e., *AX-95172478* on chromosome 1A, *AX-94875635* on 1B, *AX-94723806* on 3D, *AX-95203560* on 4A and *AX-94990952* on 6A. The same chromosomes that contain MTAs for YR resistance were also found to contain some of the significant genes linked to APR against YR, which includes *Yr16, Yr29, Yr30, Yr41, Yr49, Yr54, Yr58, Yr71*, *Yr80*, and *Yr86, * (Table 1).

The stable MTAs were used to study the effects of combinations of MTAs on YR and SR phenotype (Table 3). For SR, *AX-94883961* on 7B + *AX-94843704* on 1B, and *AX-94883961* on 7B + *AX-94580041* on 3D + *AX-94843704* on 1B were found to be superior with ACI scores of 9.45 and 9.49, respectively. These combinations were in high frequency in elite Indian genotypes and found in over 180 genotypes. Similarly, for YR, *AX-94990952* on 6A + *AX-95203560* on 4A + *AX-94723806* on 3D + *AX-95172478* on 1A, *AX-94990952* on 6A + *AX-95203560* on 4A, *AX-95203560* on 4A + *AX-95172478* on 1A were superior with ACI score of 1.36, 3.52 and 8.14 and found in very low frequency of one, two and six genotypes, respectively. This indicates that these combinations are rare and should be exploited in breeding programs using innovative ways like developing haplotypes of resistant SNPs and extensively using the genotypes with this combination in commercial breeding pipelines.

The 17 SR MTAs are distributed in 13 chromosomes with a maximum of four MTAs on 1B chromosome, followed by two MTAs each on 1D, 3D, and 4B, and one MTA each on 1A, 2B, 4A, 5B, 6A, 6B, 6D, 7D, and 7B, chromosomes. Previously, only one *Sr* gene i.e., *Sr2* [78] on the 3B chromosome was known to confer APR, now four additional genes conferring APR to SR including *Sr56* on the 5B [79], *Sr55* [80] and *Sr57* [81] on the 7D and *Sr58* [82] on the 1B chromosome have been well characterized and found useful for wheat breeding with durable rust resistance. However, the MTAs for both YR and SR resistance identified in this study are likely on different loci and have a smaller quantitative effect on imparting rust resistance. Also, none of the MTAs reported in the present study showed pleiotropy for more than one rust disease. Several MTAs were identified through GWAS or QTLs using a conventional QTL mapping approach on the same chromosomes but at different positions [45–49]. *AX-94716205* located on 1B encodes disease resistance protein (TraesCS1B02G026300) found to have a role in disease resistance crop plants. Another MTA (*AX-94,472,028*) on 3D encode ankyrin repeat (*TraesCS3D02G152600*) and its expression analysis revealed that its expression was 1.27 folds higher in stress tolerance than the control. The role of ankyrin repeat and WRKY receptors on YR resistance was reported in wheat [60]. Similarly, *AX-94878781* on 4B and *AX-94421372* on 5B encodes protein kinases (TraesCS4B02G353600, TraesCS5B02G371800) and the expression of transcript (TraesCS5B02G371800) was 1.08 folds higher. Previous studies reported the importance of major gene *Sr43* encoded protein kinase ATP binding site and *Sr60* encoded protein with two putative kinase domains in wheat that conferred significant levels of resistance to a wide range of strains of SR pathogen [61, 62]. Chromosome 4 A harbored *AX-94641391* was found to encode serine/threonine protein kinase (TraesCS4A02G353300) and has been upregulated to the tune of 2.68 folds under stress conditions compared to control conditions. Also, the role of serine/threonine kinase gene i.e., *Rpg1* in the regulation of stem rust resistance was reported in barley [63]. The expression analysis for *Sr* genes showed that, TraesCS3D02G463300 expressed 3.17 folds higher under stress conditions compared to control.

For YR field resistance, 24 MTAs were distributed in 12 chromosomes, with the largest number of four MTAs on 3B followed by three MTAs each on 2A, 2D, 3D, and 5D chromosomes. The previous reports also highlighted the importance of the 3B chromosome, as several ASR genes and three APR genes namely *Yr30/Sr2*, *Yr58*, and *Yr80* were identified on the 3B. Furthermore, previous studies also identified a few MTAs/QTLs on the same chromosomes but at different positions [35–39]. An MTA on 3B (*AX-94390305*) and 1D (*AX-94762873*) was found to encode leucine rich repeat domain super family (TraesCS3B02G587400, TraesCS1D02G002700) and expression analysis of TraesCS3B02G587400 suggested that under stress conditions it has been expressed with 0.16 TPM, however, there was no transcript expression under controlled conditions. Further, TraesCS3B02G587400 was subjected to expression network analysis to ascertain the potential link to the rust phenotype, which revealed that it is associated with the rust resistance phenotype. A few studies also highlighted the importance of nucleotide binding and leucine rich repeat (NBS-LRR) domains that confers resistance through high pathogenesis related protein (PR) productions and hypersensitive cell death. Also, the NBS-LRR protein activated by the effector protein of pathogens enables the start of the defense response like an explosion of reactive oxygen, a hypersensitive response to inhibit the pathogen growth [70]. Out of 29 cloned rust resistance genes, 23 *R* proteins belong to the NLR class [64].

The MTAs on 5D (*AX-94408063*) and on 2 A (*AX-94476121*) found to encode serine threonine/tyrosine-protein kinase (TraesCS5D02G531200, TraesCS2A02G456100) and MTA on 2A (*AX-95165557*) encodes START domain (TraesCS2A02G010200). The associated transcripts i.e., TraesCS5D02G531200, TraesCS2A02G456100, and TraesCS2A02G010200 expression was 1.37, 1.32, and 1.13 folds higher under stress conditions than control, respectively. Similarly, a kinase and a putative START lipid binding domain are integral parts of the WHEAT KINASE START1(WKS1) gene designated as a candidate for the *Yr36* gene which confers race-nonspecific resistance to YR has been cloned [71]. The thylakoid ascorbate peroxidase protein, which increases the amounts of reactive oxygen species (ROS) during immune response is thought to be phosphorylated by WKS1 [65]. WKS1 has since been demonstrated to phosphorylate the protein sbO, which is a part of photosystem II, leading to leaf chlorosis, decreased photosynthesis, and *Pst* resistance [83]. The other MTA (*AX-94448814*) on 5D encodes C2 domain family proteins (TraesCS5D02G273300). Zhang et al. [66] reported the role of C2 domain protein encoding gene i.e., TaERG3 in the regulation of YR resistance in wheat. Similarly, MTA on 2A (AX-94476121) and 1D (AX-94762873) were found to encode Zinc finger, RING/FYVE/PHD type (TraesCS2A02G456200, TraesCS1D02G002400) and TraesCS2A02G456200 was upregulated 1.36 folds under stress conditions. TaLSD1 is a wheat zinc finger protein that functions as a negative regulator of programmed cell death and contributes to wheat resistance to YR [67]. *AX-94476121* on 2A encodes Myb/SANT like domain (TraesCS2A02G455700). A novel R2R3-MYB transcription factor i.e., TaMYB29 also confers resistance against wheat YR [68]. MYB was the most abundant transcription factor in differentially expressed genes, which confers resistance in wheat against YR [69]. The maximum expression was observed for TraesCS6A02G393900 (3.02 folds) under stress conditions, followed by TraesCS3D02G530700 (1.52 folds), TraesCS1B02G337400 (1.41 folds), TraesCS4A02G498600 (1.18 folds), and TraesCS1A02G051500 (1.16) compared to control.

In the present study, we identified five stable MTAs each for SR resistance (*AX-94716205* and *AX-94843704*, *AX-94580041*, *AX-94878781*, *AX-94883961*) and YR resistance (*AX-95172478*, *AX-94875635*, *AX-94723806*, *AX-95203560* and *AX-94990952*) for use in development of rust tolerant bread-wheat cultivars. Additionally, identified the best combinations of stable MTAs for SR resistance (*AX-94883961* + *AX-94843704*, *AX-94883961* + *AX-94580041* + *AX-94843704*) and YR resistance (*AX-94990952* + *AX-95203560* + *AX-94723806* + *AX-95172478*, *AX-94990952* + *AX-95203560* and *AX-95203560* + *AX-95172478*). The elite variety, PBW827 possesses desirable MTA combinations for both YR and SR resistance and is hence useful for exploitation in rust resistance breeding. The expression analysis showed the upregulation of 18 candidate genes during rust incidence compared to rust free control. The two important candidate genes i.e., TraesCS3D02G519600 and TraesCS2D02G038900 showed expression levels 19.36 and 7.23 fold higher under stress conditions. The role of TraesCS2D02G038900 in disease resistance is further confirmed through expression network construction. The putative genes identified in the present study are targets for further validation for their role in imparting rust resistance.

## Conclusions

Most wheat growing regions have recurrent rust epidemics that cause considerable yield losses and affect grain quality, if not successfully managed. Advances in the application of genomics technologies, combined with conventional genetic and breeding approaches will help to accelerate the rate of genetic gain for rust resistance in wheat. This study identifies several stable MTAs for SR and YR resistance along with the candidate genes which can prove valuable to enhance durable rust resistance in wheat. Also, identified the best combination of MTAs for both YR and SR resistance. This may be helpful to exploit the genetic resistance through gene pyramiding. The genotype, PBW827 had the best combination of MTAs for both SR and YR resistance and could be the key donor to use in wheat breeding programs to battle ever evolving rust pathogens. The key putative candidate genes may be the important candidates for further validation and gene cloning experiments.

## Materials and methods

### Plant material and experimentation

The present experiment consists of a set of 280 diverse bread wheat genotypes, which includes advanced generation elite lines and commercial cultivars. The plant material along with pedigree details are presented in a supplementary table (Table S1). The GWAS population was phenotyped during 2019–20 (Table 5) at eight environments for SR and 10 environments each for LR and YR covering all the agro-ecological zones for wheat cultivation in India, as many of these testing sites represent the global mega-environments (MEs) [84]. The global spring wheat area was divided into six MEs, the Ludhiana location typically represents ME1 and Dharwad represents ME4 [84]. The locations present in the Himalayan region like Malan and Bajaura represent ME3. Therefore, most of the testing sites in north western plains zone including Karnal, Hisar, Pantnagar, Ludhiana, Gurdaspur, Durgapura, and Delhi may fall in ME1. Similarly, testing sites in the peninsular zone including Dharwad, Niphad, and Pune belong to ME4. The experimental genotypes were sown in two rows of one meter length with a 25 cm distance between the rows and 5 cm between the plants from 1–15th of November during 2019–20 rabi (winter) season.

**Table 5:** The list of environments and their prevailing weather parameters used for genome-wide association study population phenotyping for stem rust, leaf rust and stripe rust

Environment	Rust	Geographic coordinates	Min. Temp. (^0^C)	Max. Temp. (^0^C)	Average Temp. (^0^C)	Rainfall (mm)
Dharwad	SR, LR	15.4934°N, 74.9816°E	18.6	30.6	24.6	46
Mahabaleshwar	SR, LR	17.9378°N, 73.6730°E	15.2	27.7	21.5	362
Wellington	SR, LR	11.3796°N, 76.7738°E	12.7	21.7	17.2	330
Powarkheda	SR, LR	22.7002°N, 77.7469°E	15.0	29.8	22.4	225
Niphad	SR, LR	20.1011°N, 74.0726°E	16.0	28.3	22.2	109
Indore	SR	21.5031°N, 70.4415°E	16.1	29.7	22.9	212
Pune	SR	18.1067°N, 74.3512°E	18.0	31.6	24.8	258
Vijapur	SR	23.5708°N, 72.7513°E	14.8	27.2	21.0	116
Karnal	YR, LR	29.7050°N, 76.9924°E	13.7	25.6	19.6	364
Ayodhya	LR	26.5398°N, 81.8365°E	12.1	24.0	18.1	349
Jammu	YR, LR	32.6549°N, 74.8001°E	NA	NA	NA	NA
Hisar	YR, LR	29.1509°N, 75.6977°E	13.3	26.6	20.0	171
Pantnagar	YR, LR	29.0207°N, 79.4838°E	13.1	25.3	19.2	366
Gurdaspur	YR	32.0512°N, 75.4193°E	NA	NA	NA	NA
Durgapura	YR	26.8435°N, 75.7877°E	15.5	28.4	22.0	181
Delhi	YR	28.6398°N, 77.1584°E	13.9	26.6	20.3	50
Bajaura	YR	31.8349°N, 77.1718°E	5.9	22.3	14.1	205
Malan	YR	32.1159°N, 76.4166°E	10.8	26.1	18.4	239
Dhaulakuan	YR	30.5009°N, 77.4749°E	NA	NA	NA	NA

SR: stem/black rust; LR: leaf/brown rust; YR: yellow/stripe rust; Min. Temp. (°C): average minimum temperature during the crop growth period; Max. Temp. (°C): average maximum temperature during the crop growth period; Average Temp. (°C): average temperature during the crop growth period

.

At regular intervals of every 20 rows of test entries and as border rows, infector rows of five highly rust susceptible wheat genotypes viz., Agra Local, A-9-30-1, Malwi local, Local Wheat Hango, and HD2932 were grown. Seeds mixed in equal proportions were used to develop high disease pressure conditions for precision field phenotyping. For locations where more than one rust disease was evaluated, separate nurseries were planted maintaining sufficient isolation of more than one kilometre to avoid the confounding effect of more than one rust pathogens on rust scoring. These artificial inoculations with only one rust pathogen in the infector rows in each of the nurseries ensured the build-up of targeted rust disease on the test genotypes. There was no confounding effect of more than one rust disease. A mixture of rust inoculum of the four most prevalent and virulent pathotypes for each of the rust provided by the rust laboratory at ICAR-IIWBR Regional Station, Flowerdale, Shimla was used in each of the nurseries for developing artificial rust epiphytotic initially on infector rows and subsequently on the experimental material. Rust inocula comprised of *Pgt 40A* (62G29), *Pgt 11* (79G31), *Pgt 42−2* (58G13-3) and *Pgt 117-6* (37G19); *Pt* 77−5 (121R63-1), *Pt 77−9* (121R60-1), *Pt 104-2* (21R55) and *Pt 12−5* (29R45); *Pst 46S119*, *Pst 110S119*, *Pst 47S103*, and *Pst 110S84*.

### Phenotyping

For the inoculation of experimental material under field conditions, a mixture of urediniospores suspension in 12 L of water with three drops of TWEEN20 was sprayed at the end of December month and continued up to the first fortnight of January using an ultra low volume sprayer during the evenings with clear sky with the expectation of dew to ensure good incubation of the rust spores on the host plants. Further, infected plants in the portable pots were also kept at regular intervals in the experimentation plots to support sufficient inoculum load for disease development. The disease severity was recorded under field conditions at the adult plant stage using the modified Cobb’s scale [85]. The rust data was recorded 3–4 times and the final recording was done during the soft dough stage for YR and the hard dough stage for LR and SR. For statistical analysis, host response and rust severity data were converted into a single numerical value, which is referred to as the Coefficient of Infection (CI) [86]. To calculate CI, the disease severity was multiplied by a numerical notation for host response, where, immune = 0.0; resistant = 0.2; moderately resistant = 0.4; mixed = 0.6; moderately susceptible = 0.8, and susceptible = 1.0 [87, 88]. The CI values of all locations were averaged to calculate the ACI of each genotype for the three rust diseases. The CI of the individual location was considered as the final phenotype for the individual environment and identified the location specific MTAs; the ACI values for each rust i.e., SR, LR, and YR were used to identify average MTAs.

### Genotyping

The genomic DNA was isolated from fresh leaves of 22 day old plants by CTAB method [89]. The Axiom® Wheat Breeder’s genotyping array (Affymetrix product ID 550,524, Santa Clara, CA, USA), which contains 35,143 genome-wide SNPs was used to genotype the GWAS population of 280 lines. SNP array was developed with reference to IWGSC RefSeq assembly for *Triticum aestivum* v1.0. SNP detection is hybridization based and SNP data is obtained in HapMap format. A thorough quality check was followed by removing the SNPs that exhibited monomorphism, SNPs with less than 5% minor allele frequency (MAF), and markers with ≥ 25.0% heterozygosity, > 10% missing percentage in Microsoft excel. Thus, a set of 14,790 high quality informative SNPs were further employed for the GWAS analysis.

### Population statistics and GWAS

Trait Analysis by aSSociation Evolution and Linkage (TASSEL) program Version 5.0 [90] was used to calculate pair wise LD values. The whole genome and subgenome LD block sizes were calculated by the fixation of *r*^*2*^ threshold, where LD decayed to half of its original value [91]. The Genome Association and Prediction Integrated Tool (GAPIT) was used to estimate PCA and Kinship association [92]. We used PCA as three as it captures majority of the variation and GAPIT software itself calculated the kinship matrix from marker data and included them in the GWAS by default as co variate (GAPIT used manual v3). The genotypic data along with the corresponding phenotypic data of SR, LR, and YR was used for GWAS analysis. The principal components were included as covariates in regression models to account for population structure to reduce false positive signals. Genetic relatedness among the members of the GWAS panel is measured as Kinship in the form of a K matrix. K matrix has been fit into linear mixed models to correct the genetic variation due to relatedness. Hence, both PCA and Kinship analysis separate genetic variation due to confounding factors like population structure and genetic relatedness from true genetic effects associated with traits and hence increase the accuracy and robustness of GWAS.

The Bayesian information and Linkage Disequilibrium Iteratively Nested Keyway (BLINK) model [93] implemented in GAPIT v3.0 optimized by Wang and Zhang [94] in the R software package was used to identify the MTAs. Bonferroni correction was utilized to set an optimum *p.value* threshold to consider MTAs, as the high level of stringency applied through Bonferroni correction will reduce the false positives. To get Bonferroni correction, α parameter was set to 0.05 which was divided by the total number of SNPs (14,790), as a result, the *p.value* threshold was set at 3.38e-6. The Bonferroni corrected SNPs were applied to identify MTAs and the phenotypic variation explained (PVE) was computed.

### In silico and expression analysis

The SNP sequence of MTAs was used in BLAST with default search parameters in the Ensemble Plants database (http://plants.ensembl.org/index.html) to search putative candidate genes of the bread wheat genome [58] (RefSeq v1.0 accessed on 25th February, 2023). To identify putative candidate genes, a distance of 0.1 Mb intervals was selected at both overlapped regions and also interval regions flanked on either side of markers. The possible role of the identified candidate genes in rust resistance was also ascertained through previous reports.

The putative genes associated with MTAs were subjected to expression analysis in the Wheat Expression Browser by expVIP [95] (http://www.wheat-expression.com/). The expression of genes was recorded in the transcript per million (TPM). Searched for candidate genes in the flanking region of associated SNP using comparative genomics. Listed down the genes in the region and found out their function/protein produced from InterPro which provides functional analysis of proteins. The expression level of each transcript (genes) was obtained from the expression browser followed by a heat map to identify highly expressed genes under disease and normal conditions. The graphical representation of expression data was generated using the ‘ggplot2’ package in R software. The fold increase under stress conditions compared to the control condition is given as a supplementary table (Table S2). Additionally, potential links of highly expressed genes to phenotypes were determined using the Knetminer tool (https://knetminer.com/cereals) integrated with the Wheat Expression database.

The candidate region identified in the study may be the hotspot of genes controlling various traits. Probable genes in the vicinity of linked SNP markers can be postulated with the use of comparative genomics from available annotated genomes (IWGSC ref v1.0 in this case). Many of the genes/transcripts identified in this manner were used to screen for their level of expression using the wheat expression browser which provides detailed insights into the transcriptional landscape of bread wheat through RNA-sequencing samples alongside the annotated genome to determine the similarities and differences between homoeolog expression across a range of tissues, developmental stages, and cultivars [95]. Hence, expression analysis of the transcript (gene) in the hotspot gives an idea about the strength of the expression which can be used to identify the best gene among the pool and to avoid false associations identified in the GWAS study. These details from the expression browser can be used to find specifically adopted genes for stress and control conditions along with housekeeping genes.

### MTA’s combination effects

The stable MTAs were used to investigate their combined effect on phenotype expression. The genotypes with superior alleles for multiple stable MTAs were grouped and the average phenotype across the environments. Further, we identified the best MTA combination for superior performance to YR and SR resistance and the genotypes possessing those combinations. The stable MTAs identified in multiple environments with high phenotypic variance explained were considered for the analysis. The SNP allele linked to tolerance is selected for each MTA. Lines were screened in such a way that we grouped lines based on the different combinations of tolerant SNP alleles at different MTAs in each group. Then the combination having high tolerance to rust based on average phenotypic performance across locations is considered as best combination. This will help to identify the best donor and to prioritize the best regions for gene pyramiding and introgression.

### Electronic supplementary material

Below is the link to the electronic supplementary material.

Supplementary Material 1

Supplementary Material 2

Supplementary Material 3

Supplementary Material 4

## Data Availability

The paper and its supplementary materials contain all the data.
