# Supplementary material for: Genome-wide association study identifies novel loci and candidate genes for rust resistance in wheat (Triticum aestivum L.)
Source: BMC Plant Biol. 2024 May 17;24:411. doi: 10.1186/s12870-024-05124-2 (PMC11100168; doi:10.1186/s12870-024-05124-2)
Supplement: Supplementary file 3 — Supplementary Material 3 [file 12870_2024_5124_MOESM3_ESM.docx]

Supplementary Table 3. Rust resistant genotypes (check varieties and advanced breeding lines) with less than 5.0 average ACI (8 locations for stem rust, 5 locations each for leaf rust south and north, 11 locations for stripe rust)

| **Stem Rust + Leaf Rust (South)** | | | **Stripe Rust + Leaf Rust (North)** | | |
| --- | --- | --- | --- | --- | --- |
| SN | Genotype | Released/ Advanced breeding line | SN | Genotype | Released/ Advanced Breeding Line |
| 1 | CG1034 | Advanced breeding line | 1 | DBW316 | Released for NEPZ IR-LS |
| 2 | DBW316 | Released for NEPZ IR-LS | 2 | HI1654 | Released for CZ RI-TS |
| 3 | DBW39 | Released for NEPZ IR-TS | 3 | HS675 | Advanced breeding line |
| 4 | GW513 | Released for CZ IR-TS | 4 | JKW275 | Advanced breeding line |
| 5 | GW525 | Advanced breeding line | 5 | K1903 | Advanced breeding line |
| 6 | GW528 | Advanced breeding line | 6 | PBW813 | Advanced breeding line |
| 7 | HD2864 | Released for CZ IR-LS | 7 | PBW827 | Advanced breeding line |
| 8 | HI1544 | Released for CZ IR-TS | 8 | PBW829 | Advanced breeding line |
| 9 | HI1563 | Released for NEPZ IR-LS | 9 | PBW830 | Advanced breeding line |
| 10 | HI1634 | Released for CZ IR-LS | 10 | PBW831 | Advanced breeding line |
| 11 | HI1636 | Released for CZ IR-TS | 11 | PBW841 | Advanced breeding line |
| 12 | HI1637 | Advanced breeding line | 12 | RVW4301 | Advanced breeding line |
| 13 | HI1650 | Released for CZ IR-TS | 13 | UP3054 | Advanced breeding line |
| 14 | HI1651 | Advanced breeding line | 14 | UP3059 | Advanced breeding line |
| 15 | HI1654 | Released for NWPZ RI-TS | 15 | WH1252 | Advanced breeding line |
| 16 | HS676 | Advanced breeding line | 16 | WH1274 | Advanced breeding line |
| 17 | HS678 | Advanced breeding line | 17 | WH1278 | Advanced breeding line |
| 18 | HS679 | Advanced breeding line | 18 | WH1283 | Advanced breeding line |
| 19 | NIAW3898 | Advanced breeding line | 19 | RAJ4541 | Advanced breeding line |
| 20 | NWS2176 | Advanced breeding line | 20 | DBW318 | Advanced breeding line |
| 21 | PBW836 | Advanced breeding line | 21 | PBW835 | Advanced breeding line |
| 22 | RVW4304 | Advanced breeding line | 22 | PBW837 | Advanced breeding line |
| 23 | UAS3012 | Advanced breeding line |  |  |  |
| 24 | UP3033 | Advanced breeding line |  |  |  |
| 25 | UP3054 | Advanced breeding line |  |  |  |
| 26 | UP3058 | Advanced breeding line |  |  |  |
| 27 | UP3064 | Advanced breeding line |  |  |  |
| 28 | RAJ4541 | Advanced breeding line |  |  |  |
| 29 | HI1642 | Advanced breeding line |  |  |  |
| 30 | MACS6752 | Advanced breeding line |  |  |  |
| 31 | MACS6749 | Advanced breeding line |  |  |  |
| 32 | DBW308 | Multiple disease res. GS |  |  |  |
| 33 | DBW173 | Released for NWPZ IR-LS |  |  |  |

NEPZ IR-LS: north-eastern plain zone irrigated late sown; NEPZ IR-TS: north-eastern plain zone irrigated timely sown; CZ IR-TS: central zone irrigated timely sown; CZ IR-LS: central zone irrigated late sown; NWPZ RI-TS: north western plain zone restricted irrigation timely sown
