# Supplementary material for: Genome-wide association study identifies novel loci and candidate genes for rust resistance in wheat (Triticum aestivum L.)
Source: BMC Plant Biol. 2024 May 17;24:411. doi: 10.1186/s12870-024-05124-2 (PMC11100168; doi:10.1186/s12870-024-05124-2)
Supplement: Supplementary file 4 — Supplementary Material 4 [file 12870_2024_5124_MOESM4_ESM.docx]

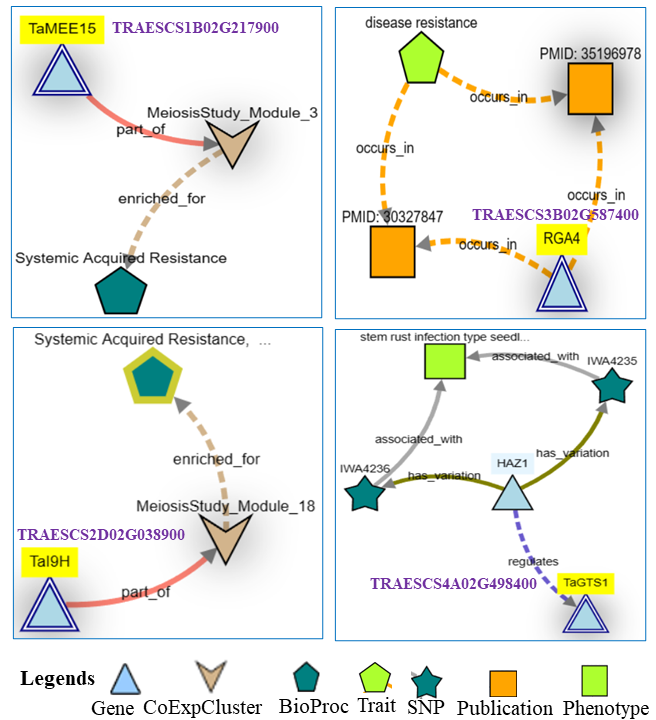


**Supplementary Fig. 1** Expression networks of highly expressed genes in imparting rust resistance. BioProc is Biological process, CoExpCluster is Co-expression clusters.
